# RETRACTION: Computational and Preclinical Evidence of Anti‐ischemic Properties of L‐Carnitine‐Rich Supplement via Stimulation of Anti‐inflammatory and Antioxidant Events in Testicular Torsed Rats

**DOI:** 10.1155/omcl/9793178

**Published:** 2026-07-10

**Authors:** Oxidative Medicine and Cellular Longevity

RETRACTION: J. O. Olugbodi, K. Samalia, B. Lawal, et al., “Computational and Preclinical Evidence of Anti‐ischemic Properties of L‐Carnitine‐Rich Supplement via Stimulation of Anti‐inflammatory and Antioxidant Events in Testicular Torsed Rats,” *Oxidative Medicine and Cellular Longevity*, vol. 2021 (2021). https://doi.org/10.1155/2021/5543340.

The above article, published online on 5 July 2021 in Wiley Online Library (wileyonlinelibrary.com), has been retracted following the agreement of the Journal’s Editor‐in‐Chief, Jeannette Vasquez‐Vivar; and John Wiley & Sons Ltd.

The retraction has been agreed following an investigation of the concerns flagged by a third party on PubPeer [1]. More specifically, concerns were found relating to the majority of the images shown in Figures 5–8, with multiple overlapping features identified.

The authors were contacted to respond to the above concerns and were unable to verify the provenance, integrity, and experimental group assignment of the supplied histopathology panels.

As a result of the investigation, the data and conclusions of this article are considered unreliable and the article is therefore retracted.

The authors disagree with this retraction.
